# Identification of *Akkermansia muciniphila* as a candidate gut commensal involved in the pathogenesis of rheumatoid arthritis

**DOI:** 10.3389/fimmu.2026.1917757

**Published:** 2026-10-01

**Authors:** Lucy M. Curham, Korawit Kanjana, Cameron A. Deangelo, David E. Kling, Maissa M. Gaye, Yi Yang, Chaoshuang Xia, Sheila Arvikar, Noah W. Palm, Catherine E. Costello, James J. Moon, Allen C. Steere

**Affiliations:** 1 Center for Immunology and Inflammatory Diseases, Division of Rheumatology, Allergy, and Immunology, Massachusetts General Hospital, Harvard Medical School, Boston, MA, United States; 2 Center for Biomedical Mass Spectrometry, Boston University Chobanian and Avedisian School of Medicine, Boston, MA, United States; 3 Department of Immunobiology, Yale University School of Medicine, New Haven, CT, United States

**Keywords:** autoimmunity, B cell, mucosal origins hypothesis, rheumatoid arthritis, T cell

## Abstract

**Objective:**

The “mucosal origins hypothesis” posits that mucosal sites, such as the gut, are initial sites where immune responses first develop that may, months to years later, result in clinical rheumatoid arthritis (RA). Our goal here was to conduct a discovery-based, hypothesis-generating study using samples from a relatively small number of patients to implicate previously unrecognized highly IgA-coated commensal flora that may have a role in RA pathogenesis (primary outcome). The secondary supporting outcomes were to determine whether HLA-DR-presented microbial peptides from such flora could be identified in synovial tissue and to determine T and B cell responses to these peptides and citrullinated proteins.

**Methods:**

Highly IgA-coated bacteria taxa were identified in RA patients’ stool samples by 16s rRNA sequencing; HLA-DR-presented peptides derived from the most common, highly IgA-coated microbes were identified from synovial tissue by liquid chromatography–tandem mass spectrometry; T cell responses to the identified microbial and human mimic epitopes were determined by IFNγ ELISpot assays, and citrullinated IgG and IgA antibodies to implicated microbial and human proteins were measured by ELISA.

**Results:**

Of 32 RA patients, 28 (88%) had highly IgA-coated *Akkermansia muciniphila* (*Am*) in stool samples. HLA-DR presentation of peptides derived from two *Am* proteins, including the molecular chaperone DnaK protein (*Am*-cDnaK), was identified from RA synovial tissue, suggesting that the microbe or its components may reach the joints. Eight of 21 patients (38%) had T cell responses to the *Am*-cDnaK peptide and a human heat shock protein-mimic peptide (Hu-HSPA5) and/or IgG antibodies to citrullinated forms of these proteins. Reactivity was found primarily in pre-RA and early RA soon after transition to clinical RA and less commonly during later flares of arthritis.

**Conclusions:**

These data implicate *Am* as a candidate gut commensal involved in RA pathogenesis and may serve as a compass for planning larger studies to test the hypothesis that *Am*, as well as certain other highly IgA-coated commensal flora or their components, may sometimes reach joints where they initiate or enhance autoimmune T cell responses via epitope mimicry and epitope spreading, leading to antibody responses to citrullinated host proteins in joints.

## Introduction

Rheumatoid arthritis (RA), a chronic inflammatory form of polyarthritis, is propagated by both genetic and environmental factors (1). Presentation of antigens to Th1 cells by certain HLA-DR molecules with a shared epitope (SE) and the development of anti-citrullinated protein antibodies (ACPA) are key markers of the disease (2, 3). Increasing evidence points to the “mucosal origins hypothesis” as initial sites in which these immune responses first develop, particularly in the gut, periodontium, or lung, often several years before the appearance of clinical RA (4, 5). To date, cigarette smoking (6, 7), several organisms associated with periodontitis (8–10), and two commensal microbes in the gut (11, 12) have been postulated to trigger such immune responses.

According to the mucosal origins hypothesis, the initial step is immune responses to bacterial proteins in the mucosa, including citrullinated bacterial proteins (13). Because of epitope mimicry and epitope spreading, these responses spread to citrullinated self-proteins. Why these antibodies then target the joints is less clear. One postulate is that mucosal immune responses to certain commensal microbes include cross-reactive antibodies to citrullinated microbial and human proteins that may also target human self-proteins in joints. A modification of this hypothesis is that certain commensal organisms or their components may reach joints where they ignite a dysregulated proinflammatory immune response (14), which includes epitope mimicry and epitope spreading to these microbial proteins and to additional human proteins.

In 2013, Scher et al. reported that *Prevotella* spp., especially *P. copri*, was overexpanded in stool samples from patients with new-onset RA (NORA) (15). In our initial study, using nano-liquid chromatography–tandem mass spectrometry (nano-LC–MS/MS), we identified from the PBMC of one RA patient an HLA-DR-presented peptide derived from a 27-kD secreted protein of *P. copri* (*Pc*-p27) (11). In addition, we identified a synovial HLA-DR-presented peptide from an apparently citrullinated self-protein, *N*-acetylglucosamine-6-sulfatase (GNS), which has marked sequence homology with sulfatase proteins of *Prevotella* species (16). T and B cell responses to *Pc-*p*27* and to citrullinated human GNS were found in 42% and 32% of patients, respectively, and in 3 of 18 RA patients, *Prevotella* DNA was identified in synovial fluid (11). Additionally, serum samples from the multi-center Etiology of Rheumatoid Arthritis cohort suggested a potential etiologic role for *P. copri* in the preclinical evolution of RA (17). As proof of concept, other investigators showed that one strain of *P. copri* carrying a disease-related transposon enhanced joint inflammation in two mouse models of arthritis (18). Thus, identification of HLA-DR-presented *P. copri* and human mimic peptides from PBMC or synovial tissue, detection of *Prevotella* DNA in synovial fluid, and determination of T and B cell responses to implicated human and self-peptides or proteins support the hypothesis that *P. copri* or its components may sometimes reach joints, leading to or enhancing an inflammatory immune response there.

The most recent information about the role of commensal gut flora in RA pathogenesis was the identification of *Subdoligranulum didolesgii* (*S. dido*), a member of the *Ruminococcus* family, as a potential pathobiont in RA patients (12). The organism was initially identified in stool samples using monoclonal antibodies derived from IgA and IgG double-positive plasmablasts obtained from individuals at risk for RA. One isolate tested, but not another, significantly activated CD4+ T cells in RA patients, and mice mono-colonized with this isolate developed arthritis with features reminiscent of RA. Thus, this experience showed that one commensal may be sufficient to trigger joint inflammation, but taken together, the *P. copri* and *S. dido* examples suggest that there may be multiple gut microbes capable of promoting RA progression.

We report here the identification of a third gut commensal, *Akkermansia muciniphila*, as a candidate gut commensal involved in RA pathogenesis. In our study, this microbe was one of the most highly IgA-coated in stool samples in RA patients (primary outcome); an *Am* HLA-DR-presented peptide (*Am*-cDnaK) was identified from RA patient synovial tissue and synovial fluid mononuclear cells (SFMC), and a subset of patients had T cell responses to the implicated *Am* peptides and human mimic peptides and/or antibody responses to citrullinated proteins derived from these peptides (secondary supporting outcomes). Thus, as with *P. copri*, these data support the hypothesis that *Am* or components of it may reach joints where they may initiate or enhance joint inflammation.

## Materials and methods

### Patients and healthy control subjects

The 32 adult patients with RA (age 18 or older) met the 2010 criteria of the American College of Rheumatology (ACR) and the European League Against Rheumatism (EULAR) for the diagnosis of RA (19). We have previously published the inclusion and exclusion criteria for our RA study patients (20). These studies were approved by the Human Investigations Committee at Massachusetts General Brigham Hospital. A comprehensive workflow scheme is shown in **Figure 1**. Strengthening the Reporting of Observational Studies in Epidemiology (STROBE) recommendations were observed in the reporting of this study (21).

**Figure 1:**
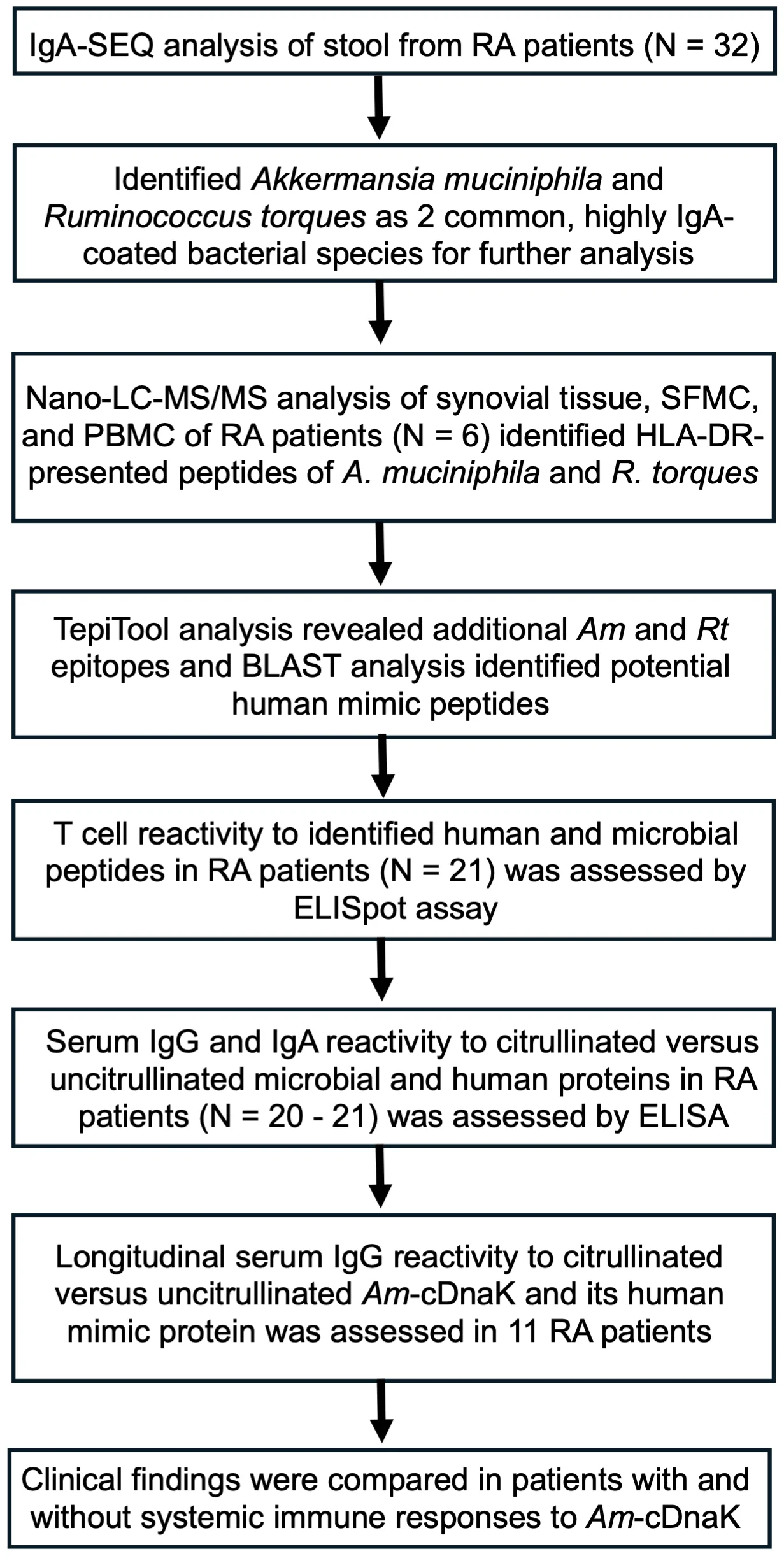
Workflow schematic of the study. This figure provides the major steps taken in carrying out this study.

The study design was a discovery-based, hypothesis-generating, retrospective, case–control study based on RA patients referred to two study physicians (ACS and SA) in the Rheumatology Clinic at Massachusetts General Hospital (MGH). Between July 2017 and December 2019, 52 consecutive patients from New England, primarily Massachusetts, were enrolled in the study, and 32 (62%) produced analyzable stool samples, which was required for inclusion in the study. The 20 patients who were not included either found it difficult to produce the sample or it was degraded by the time it reached the laboratory. Based on previous experience with *P. copri* (11), we estimated that analyzable samples from 30 patients would be sufficient to provide an initial, discovery-based analysis of RA patients’ commensal flora (the primary outcome).

At the first visit, after informed consent was obtained, blood was collected for PBMC isolation, HLA typing, and serum antibody studies. In addition, the patients were given a kit to collect a stool sample at home, with the sample returned to our laboratory by FedEx within 24 h of collection. Cryo-preserved synovial tissue and/or SFMC was reanalyzed from six additional patients with chronic RA (CRA) who had a synovectomy or total joint replacement. Serum samples were cryopreserved in freezers at -80 °C, and PBMC, SFMC, and synovia were cryopreserved in liquid nitrogen freezers at -140°C. The patients were treated with disease-modifying anti-rheumatic drugs (DMARDs), most commonly with methotrexate and, if needed, with TNF inhibitors, according to the guidelines of the ACR (19). For healthy control subjects, PBMC and serum samples for T and B cell studies were obtained from laboratory personnel. Serum samples from laboratory personnel and de-identified samples from MGH blood bank donors were used for antibody analyses.

### IgA-Seq and 16S rRNA gene sequencing

Identification of IgA-coated intestinal bacteria in the feces of RA patients was performed as previously described (22). Briefly, homogenized fecal material was centrifuged, and the supernatant was stained with PE-conjugated anti-human IgA prior to magnetic sorting of IgA-positive and IgA-negative bacteria. V4 16S rRNA gene sequencing was then performed on the bacteria in the IgA-positive and IgA-negative fractions as described previously using 2×250 sequencing on an Illumina MiSeq (23). An IgA coating index (ICI) was calculated as the ratio of the IgA-coated fraction divided by the sum of the IgA-coated and IgA-uncoated fractions. Technical duplicates were analyzed for each sample, but for some low-abundance taxa, only one value was attainable. Data are presented here on a log scale.

### Isolation and identification of HLA-DR-presented peptides

Detailed methods for the isolation and identification of HLA-DR-presented peptides from synovial tissue, SFMC, and PBMC have been previously published (24). Briefly, immunoaffinity purification was performed on lysed homogenates of synovial tissue (8–10 g), SFMC, or PBMC. The HLA-DR-presented peptides were eluted in 0.5% formic acid, separated using an Acquity M-class UPLC system (Waters) and analyzed using the Fusion LUMOS Tribrid Orbitrap mass spectrometer (Thermo Fisher Scientific) in combination with a nanoelectrospray ion source (Triversa Nanomate, Advion Interchim Scientific). Because microbial mass spectra databases are not available, human UniProt-derived peptide databases in unspecific digestion mode were used to generate *Am* and *Rt* protein databases. MS/MS spectra derived from patient samples were evaluated against the UniProt-derived peptide databases. Tandem mass spectra were analyzed using Peaks Studio X+ software (Bioinformatics Solutions Inc), and assignments were verified by manual inspection.

### ELISpot T cell assay

HLA-DR-presented and HLA-DR-predicted peptides were synthesized (GenScript), and their immunogenicity was determined by stimulation of PBMC from RA patients or healthy controls in IFNγ enzyme-linked immunospot (ELISpot) assays (Cellular Technology Ltd.). Cryopreserved PBMC from patients and controls were thawed and rested overnight. PBMC were seeded at 2 × 10^5^ cells per well in supplemented RPMI 1640 medium and stimulated in duplicate with individual peptides (2 μM per peptide) for 4 days at 37 °C with 5% CO_2_. The cells were then transferred to ELISpot plates coated with anti-IFNγ antibodies and cultured overnight. Spot-forming units (SFU) were imaged using an ImmunoSpot series 3B analyzer (Cellular Technology Ltd.) and counted using ImmunoSpot software.

### *In vitro* citrullination assay

Recombinant preparations of *Am-*cDnaK, *Rt-*cDnaK, and human HSPA5 proteins were made by GenScript. The purity of recombinant *Am-*cDnaK, *Rt-*cDnaK, and human HSPA5 was estimated to be ≥85%, ≥60%, and ≥90%, respectively, as determined by SDS-PAGE under reducing conditions.

Recombinant proteins (1 mg/mL) were incubated with a recombinant human/mouse PAD cocktail protein (SinoBiological USA) at an enzyme-to-protein ratio of 1:25. The reaction was performed in a citrullination buffer containing 100 mM Tris-HCl (pH 7.5), 10 mM CaCl_2_, and 5 mM DTT at 37 °C for 15 h. A negative control reaction was performed, substituting 10 mM EDTA for 10 mM CaCl_2_. Protein citrullination was confirmed by anti-modified citrulline immunoblotting, according to the manufacturer’s instructions (Sigma-Aldrich).

### ELISA for serum IgG and IgA antibody determinations

Medium binding ELISA plates (Immulon) were coated with recombinant proteins diluted to 1 μg/mL for IgG detection or to 2 μg/mL for IgA detection in 1X coating buffer (Candor BioScience) overnight at 4 °C. The plates were then blocked, followed by loading of diluted serum samples (1:25 for IgA detection and 1:50 for IgG detection) in duplicate wells and incubated at 37 °C for 45 min. After washing, the plates were then incubated with horseradish peroxidase (HRP)-conjugated goat anti-human IgG (1:2,000; Jackson ImmunoResearch) or HRP-conjugated goat anti-human IgA (1:1,000; Jackson ImmunoResearch) at 37 °C for 45 min. Tetramethylbenzidine (TMB) substrate solution was then added, and the reaction was terminated by the addition of 1 M H_2_SO_4_. The absorbance was read at 450 nm to determine the OD_450_ values.

### Statistics

Quantitative data were analyzed using Mann–Whitney *U*-test, and categorical data were analyzed by using Fisher’s exact test. All analyses were performed using GraphPad Prism (version 10.6.1 for Mac; GraphPad Software, Boston, MA, USA). For ELISA and ELISpot assays, the participants’ samples were assayed in technical duplicate, with the dots on plots representing the mean value of the technical duplicates for each participant. All *P*-values are two-tailed. *P*-values less than or equal to 0.05 were considered statistically significant.

## Results

### Identification of highly IgA-coated microbiota in stool samples from RA patients

To identify immunostimulatory intestinal bacteria in RA patients, we performed IgA-SEQ on fecal samples from 32 patients; 16 had new-onset RA (NORA) and 16 had chronic RA (CRA). An IgA coating index (ICI) was calculated as the ratio of the IgA-coated fraction divided by the sum of the IgA-coated and IgA-uncoated fractions and presented on a log scale.

As determined by 16S sequencing, a large number of highly IgA-coated bacterial species (ICI ≥ 0.7) were identified in the stool samples (**Figure 2**). Because patient samples were limited for subsequent testing, we chose two of the most common, highly IgA-coated microbes, *Am* and *Ruminococcus torques* (*Rt*), for further study. *Am* was highly IgA-coated in 13 of the 16 NORA patients and 15 of 16 CRA patients, whereas *Rt* was highly IgA-coated in the stool fraction of all 16 NORA patients and in 14 of the 16 CRA patients. Since the results were similar in NORA and CRA groups, data for the two groups were combined for presentation here (**Figure 2**). Taken together, *Am* was highly IgA-coated in samples from 28 of the 32 patients (88%) (median ICI value = 1.00 [range of detectable values, 0.74–1.00]), with the maximum ICI value (1.00) observed in 16 of the 32 patients (50%). Similarly, *Rt* was highly IgA-coated in 30 of the 32 patients (94%) (median ICI value, = 1.00 [range of detectable values, 0.14–1.00]), with a maximum ICI score detected in 25 of the 32 patients (78%).

**Figure 2:**
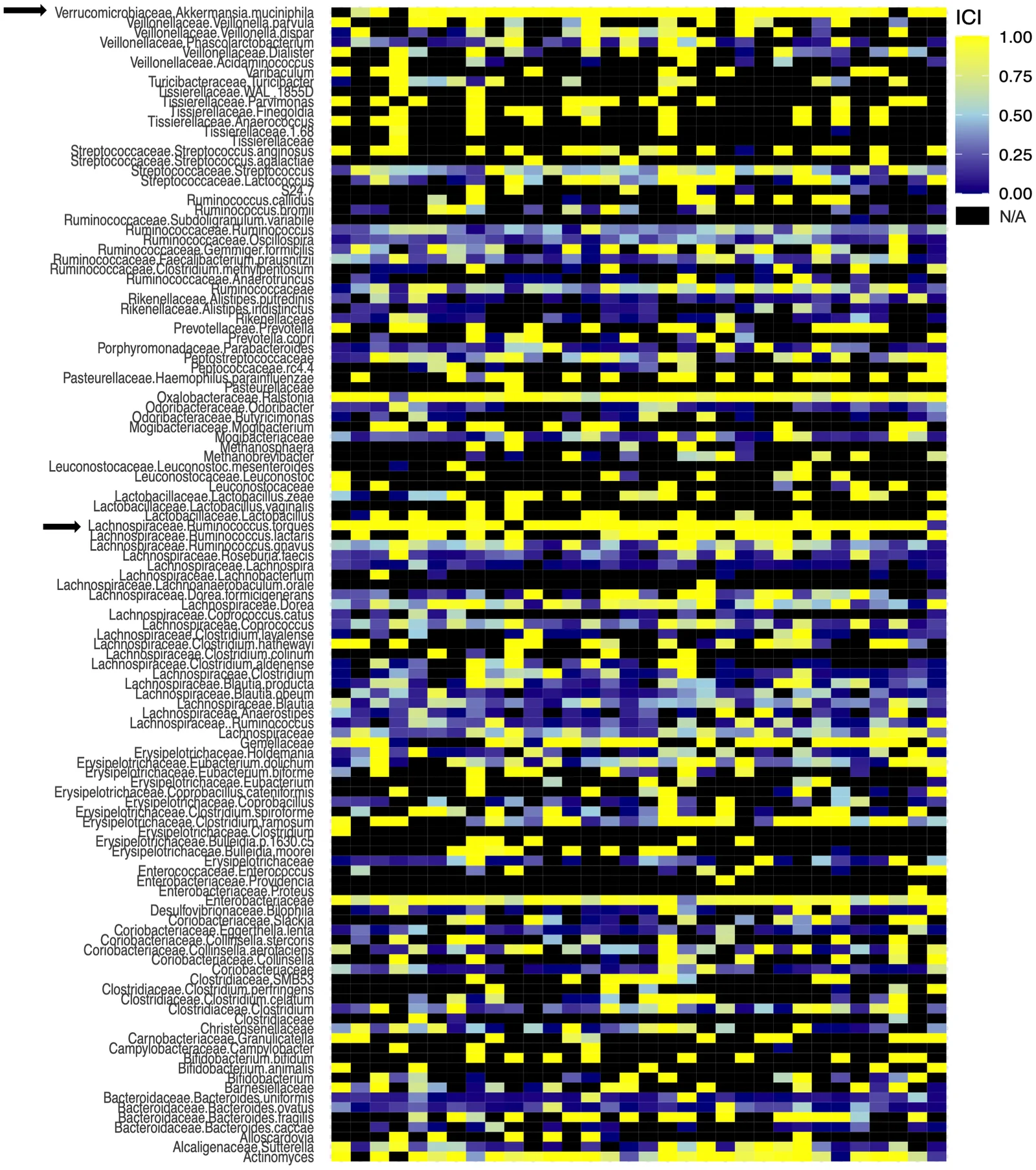
Identification of highly IgA-coated microbiota in stool samples from RA patients. Heatmap depicting the IgA coating index (ICI) scores of 117 taxa in stool samples of 32 RA patients. Each column represents an individual subject. Unattainable values are represented by black boxes (N/A, not available). Black arrows identify *Akkermansia muciniphila* and *Ruminococcus torques*.

### Identification of HLA-DR-presented microbial peptides from synovial tissue, SFMC, or PBMC of RA patients

Using nano-LC–MS/MS to identify HLA-DR-presented peptides, we previously published the self-peptides identified from synovial tissue, SFMC, or PBMC from five RA patients (24). For the current study, large-enough synovial tissue, SFMC, or PBMC samples remained for retesting from four of the five previous RA patients (RA2 through RA5). These samples, in addition to SFMC and/or PBMC from two recent RA patients (designated RA6 and RA8), were analyzed with the more recently available, highly sensitive Fusion LUMOS tandem mass spectrometer. Using the UniProt sequence database, a predicted MS/MS spectral library was assembled in-house for *Am* and *Rt* to facilitate peptide assignments for the MS/MS spectra and to predict the protein of origin for each peptide.

With these database searches, the sequences of two microbial HLA-DR-presented peptides were identified. From the synovial tissue of one patient (RA5), an HLA-DR-presented peptide (LPQAFAKMGSSVVFGS) had 100% sequence identity with an *Am* sodium/calcium symporter (*Am*-SCS) protein (**Figure 3A**). The sequence of a second HLA-DR-presented peptide (RIINEPTAAALAYG) was assigned from the synovial tissue of patient RA3 who had the RA shared epitope (RA SE) DRB1*0401 allele and from the SFMC of patient RA4 who also had the RA SE DRB1*0401 allele (**Figures 3B, C**). This peptide had 100% sequence identity with the *Am* molecular chaperone protein DnaK (*Am*-cDnaK). In addition, the identical underlined nine-amino-acid (aa) core sequence was assigned to a molecular chaperone protein DnaK and a heat shock protein 70 in the *Rt* database (**Table 1**). Although the *Am* and *Rt* peptides differed at the P-2 flanking region of the epitope, we included both the *Am*-cDnaK and *Rt*-cDnaK peptide sequences in our T cell testing of HLA-DR-presented synovial peptides.

**Figure 3:**
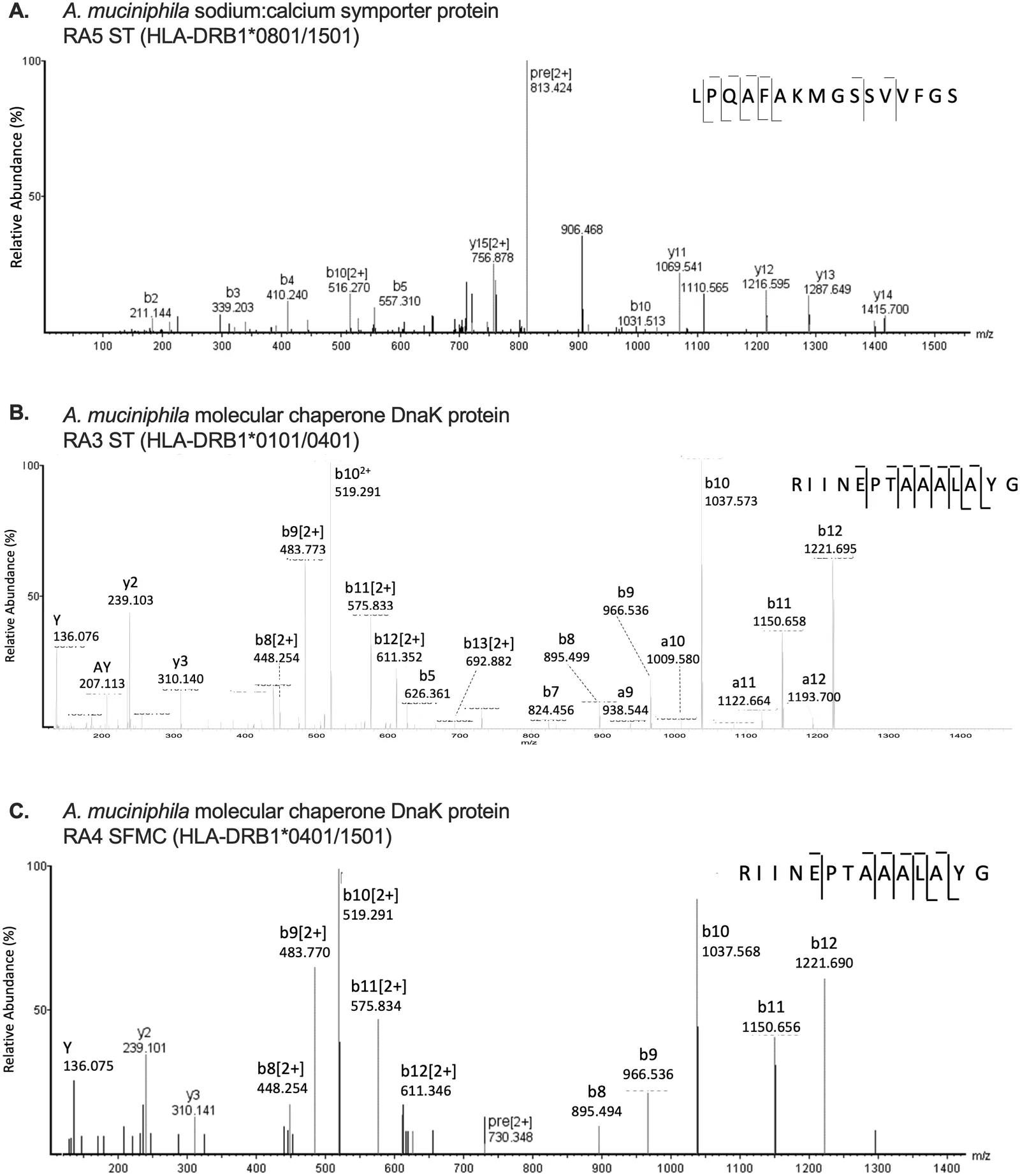
Mass spectra for HLA-DR-presented microbial peptides from synovial tissue or synovial fluid mononuclear cells (SFMC) of RA patients identified by nano-LC–MS/MS. From this MS/MS spectrum obtained during the analysis of the synovial tissue (ST) of RA5, the peptide LPQAFAKMGSSVVFGS from the *Am* sodium/calcium symporter protein was identified **(A)**. The peptide RIINEPTAAALAYG from the *Am* molecular chaperone DnaK protein was identified from the MS/MS spectrum obtained during the analysis of ST of RA3 **(B)** and from a virtually identical MS/MS spectrum obtained during the analysis of SFMC of RA4 **(C)**.

The TepiTool algorithm (version Consensus 2.22), which is featured in the epitope prediction tools of the Immune Epitope Database (IEDB) (25), was then used to search for other candidate T cell epitopes derived from these two microbial proteins. Four additional predicted epitopes were identified in the sequence of the *Am-*SCS protein, two additional epitopes were identified in the *Am-*cDnaK protein, and two were identified in the *Rt* heat shock protein (*Rt*-HSP70) (**Table 1**). Thus, 11 microbial peptides were altogether identified directly from the patients’ samples or by TepiTool.

**Table 1:** Immunogenic HLA-DR-presented and HLA-DR-predicted microbial peptides and self-peptides.

Peptide Group	Peptide no.	Protein Name	Accession no.	Species	Identification method	Sequence^+^
1	A1	Sodium:calcium symporter	PNC64835.1	*Am*	**MS/MS of synovia**	^396^LPQA**F**A**KMGSSV**VFGS^411^
	H1	Polymerase kappa isoform 3	AAV80829.1	Human	BLAST	^129^RRY**F**I**KMGSSV**ENDN^143^
2	A2	Sodium:calcium symporter	PNC64835.1	*Am*	TepiTool, predicted	^16^EA**FFVS**I**PS**MS**S**S**K**A^30^
	H2	Transient receptor potential cation channel subfamily M member 7	NP_060142.3	Human	BLAST	^1399^TK**FFVS**T**PS**QP**S**C**K**S^1413^
3	A3	Sodium:calcium symporter	PNC64835.1	*Am*	TepiTool, predicted	^09^FGSLF**FLLLTVA**AVTSSI^426^
	H3	Solute carrier family 38 member 6 isoform X9	XP_011534771.1	Human	BLAST	^77^GFS**FLLLTVA**LLASY^91^
4	A4	Sodium:calcium symporter	PNC64835.1	*Am*	TepiTool, predicted	^216^FM**PLLLLI**S**LVL**LVRI^231^
	H4	Cystatin-like 1	AAI17397.1	Human	BLAST	^8^N**PLLLLI**A**LVL**SAKL^22^
5	A5	Sodium:calcium symporter	PNC64835.1	*Am*	TepiTool, predicted	^572^YAL**FLIF**I**TM**ASGRHKVY^589^
	H5	Galanin receptor type 2 isoform X1	XP_011523729.1	Human	BLAST	^43^AVH**FLIF**L**TM**HASSF^57^
6	A6	Molecular chaperone DnaK	WP_012420444.1	*Am*	**MS/MS of synovia**	^167^**V**R**RIINEPTAAA**L**AYG**^182#^
	H6*	HSPA5 protein	AAI12964.1	Human	BLAST	^196^**V**M**RIINEPTAAA**I**AYG**^211^
7	A7	Molecular chaperone DnaK	WP_012420444.1	*Am*	**MS/MS of synovia**	^168^R**RIINEPTAAALAYG**^182#^
	H7.1	HSPA5 protein	AAI12964.1	Human	BLAST	^197^M**RIINEPTAAA**I**AYG**^211^
	H7.2	HSPA9 protein, partial	AAH30634.1	Human	BLAST	^219^L**R**V**INEPTAAALAYG**^233^
8	A8	Molecular chaperone DnaK	WP_012420444.1	*Am*	TepiTool, predicted	^384^N**DVLLLDVTPL**T**LSIE**^399^
	H8.1*	HSPA9 protein, partial	AAH30634.1	Human	BLAST	^435^T**DVLLLDVTPL**S**LGIE**^450^
	H8.2	Heat shock cognate 71 kDa protein isoform 1	NP_006588.1	Human	BLAST	^389^Q**D**L**LLLDVTPL**S**LGIE**^404^
9	R1	Molecular chaperone DnaK	WP_423248724.1	*Rt*	**MS/MS of synovia**	^141^**V**K**RIINEPTAAA**L**AYG**^156#^
		Heat shock protein 70	CUM99755.1	*Rt*	**MS/MS of synovia**	^141^**V**K**RIINEPTAAA**L**AYG**^156#^
	H6*	HSPA5 protein	AAI12964.1	Human	BLAST	^196^**V**M**RIINEPTAAA**I**AYG**^211^
10	R2	Heat shock protein 70	CUM99755.1	*Rt*	TepiTool, predicted	^360^GE**VLLLDVTPLSLSIE**^375^
	H8.1*	HSPA9 protein, partial	AAH30634.1	Human	BLAST	^435^TD**VLLLDVTPLSLGIE**^450^
11	R3	Heat shock protein 70	CUM99755.1	*Rt*	TepiTool, predicted	^427^**LGQF**R**L**D**GIPPA**R**RG**^441^
	H9	HSPA9 protein, partial	AAH30634.1	Human	BLAST	^502^**LGQF**T**L**I**GIPPA**P**RG**^516^

Predictions were made using TepiTool (Consensus v2.22) from the IEDB. The asterisk (*) identifies repeated peptides.

^a^The predicted binding core in the peptide sequence is underlined and identical amino acid in each peptide group are shown in bold.

^b^Since the sequence identified by nano-LC–MS/MS was 14 amino acids, additional one or two amino acids were added based on the known sequence of the protein.

Basic Local Alignment Search Tool (BLAST) searches revealed eight candidate *Am-*self-peptide-mimic pairs and three *Rt*-self-peptide-mimic pairs (**Table 1**). For the *Am*-SCS peptide, the human mimic pairs (peptide groups 1 through 5) shared between six or seven aa residues in the predicted nine-aa binding groove of shared epitope HLA-DR molecules. The cDnaK-self-peptide mimic pairs had identical predicted HLA-DR binding cores (nine of nine aa) in both the *Am-*cDnaK peptide and in the self-peptides identified from human heat shock proteins HSPA5 and HSPA9, and in the heat shock cognate 71-kDa protein. The closest match, *Am*-cDnaK peptide and the human mimic Hu-HSPA5 peptide, differed only in the P-2 and P10 flanking regions (peptide group 6; **Table 1**). A similar sequence homology was also observed for two of the three *Rt*-self-peptide-mimic pairs (peptide groups 9 and 11; **Table 1**).

Using the Consensus 2.22 prediction, the 11 peptides derived from *Am* or *Rt* and their human mimic peptides were predicted to be strong binders to HLA-DR molecules encoded by RA SE alleles (HLA-DRB1*0101, *0102, *0401, *0404, *0405, *0408, *1001) (rank score < 10), as well as to certain other DRB1 alleles (**Figure 4**). Thus, *Am*-SCS, *Am*-cDnaK, and *Rt*-HSP70 were predicted to be strongly bound by RA SE molecules, but a number of other DRB1 molecules were also predicted to bind these peptides.

**Figure 4:**
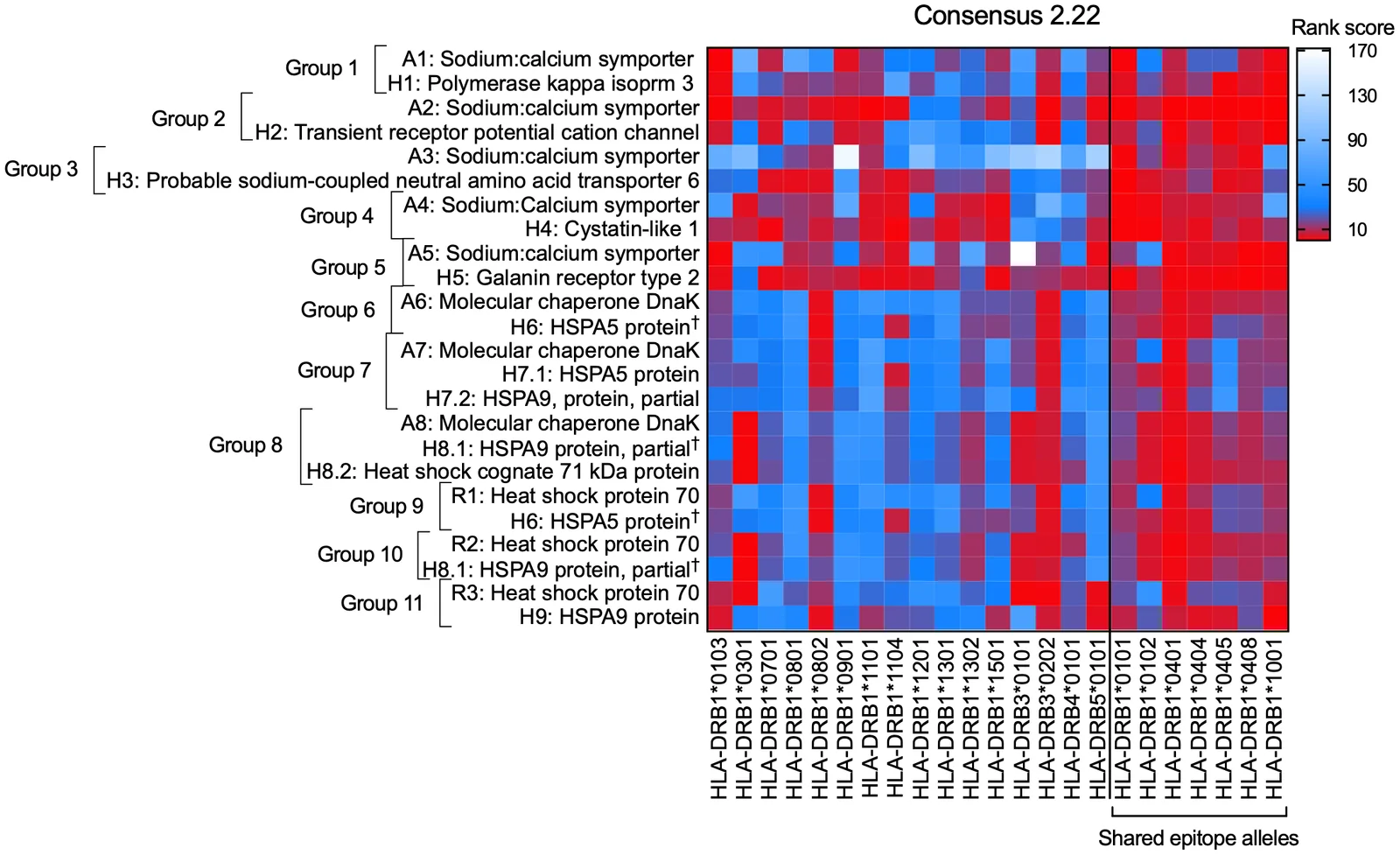
Predicted binding of *Am* and *Rt* peptides and their human mimic peptides to HLA-DR molecules. Heatmap of adjusted percentile ranks demonstrating the predicted binding of *Am* and *Rt* peptides and their corresponding human mimic peptides by 23 HLA-DR molecules, including RA shared epitope HLA-DRB1 molecules. The adjusted percentile ranks represent the relative binding affinity of a peptide to a given major histocompatibility complex (MHC) molecule with a lower percentile rank (dark red color), indicating stronger predicted binding (less peptide needed to bind the MHC). Rank values were predicted using the IEDB database (Consensus 2.22). ^†^identifies repeated peptides.

### T cell reactivity to microbial and human mimic peptides

Of the 32 patients with fecal IgA-SEQ analyses, 21 had PBMCs available for further analysis of T cell reactivity by IFNγ ELISpot assay (**Figure 5**). Cells from the 21 patients were stimulated with synthesized HLA-DR-presented peptides derived from the *Am-*SCS, *Am*-cDnaK, or *Rt*-HSP70 proteins or with their predicted human mimic peptides. Positive (anti-CD3/CD28 stimulation) and negative (medium only) controls were included in each assay.

**Figure 5:**
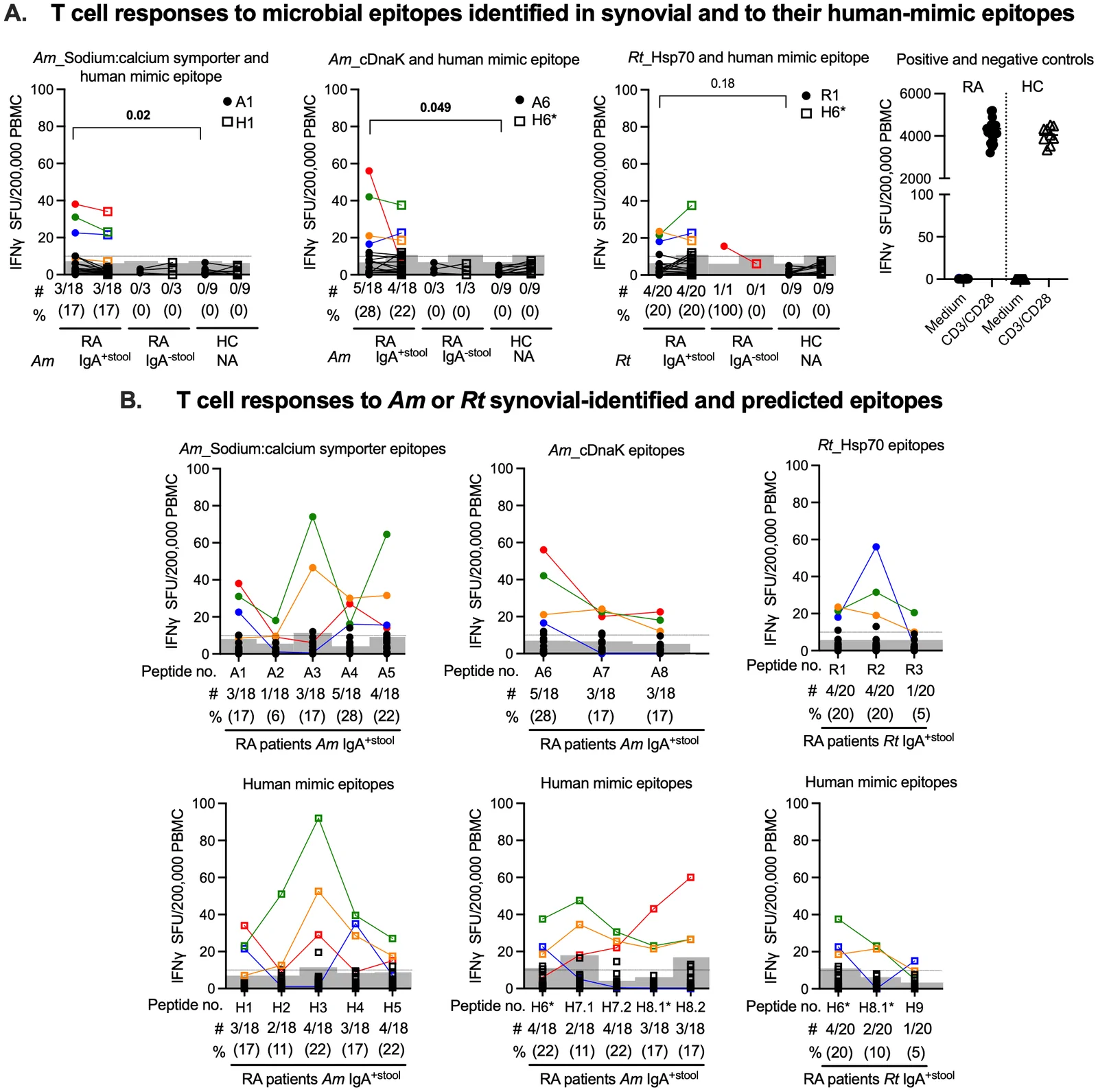
T cell reactivity to identified and predicted microbial and human mimic epitopes. PBMC from RA patients (*n* = 21) and healthy control subjects (*n* = 9) were stimulated with *Am* peptides (A1, A6) identified in synovia, a predicted *Rt* peptide (R1), or their corresponding human mimic peptides (H1, H6) [**(A)** left and middle]. Positive (anti-CD3/CD28 Dynabeads) and negative (medium alone) controls were also included [**(A)** right]. PBMC from RA patients with IgA-coated *Am* and/or *Rt* in stool samples were stimulated with additional predicted *Am* or *Rt* peptides (A1–A8, R1–R3) [**(B)** top] or with corresponding human mimic epitopes (H1–H9) [**(B)** bottom]. The HLA-DR alleles of the four patients with T cell reactivity were as follows: red dot—DRB1*0404/1104, blue dot—*0404/0701, orange dot—*0101/0101, and green dot—*1104/1301. The IFNγ levels were determined by ELISpot assay; a positive response was defined as a value >3 SD above the mean value for healthy controls (area above the gray-shaded region). If the mean value was <10 SFU, a value of 10 SFU was used (dashed line). The asterisk (*) identifies repeated peptides.

Of the 21 patients, 18 had highly IgA-coated *Am* in stool samples (IgA^+stool^). PBMC from three of these patients (17%) had positive T cell responses to the HLA-DR-presented epitope of the *Am-*SCS protein identified in synovial tissue (A1 peptide, **Figure 5A**, left). The three patients and one additional patient also had reactivity with one or more of the four other *Am*-SCS epitopes predicted from the IEDB database (A2–A5 peptides, **Figure 5B**, left upper panel). Additionally, these patients had reactivity with two or more of the five corresponding human mimic epitopes (H1–H5 peptides, **Figure 5B**, left lower panel). None of the three RA patients with IgA^-stool^ or HC subjects had positive responses to these *Am*-SCS or human mimic epitopes (**Figure 5A** and data not shown).

Of the 18 patients with *Am*-IgA*^+^*^stool^ samples, PBMC from five patients (28%) had T cell responses with the HLA-DR-presented *Am-*cDnaK epitope identified in synovial tissue and in SFMC (A6 peptide, **Figure 5A**, middle). Additionally, three of these patients had responses to other *Am*-cDnaK epitopes (A7–A8 peptides), and two to four of these patients had reactivity with corresponding human mimic epitopes (H6–H8 peptides, **Figure 5B**, middle). In contrast, the patients with *Am*-IgA*^-^*^stool^ samples and HC subjects lacked responses to these predicted microbial or human epitopes (data not shown).

Almost all RA patients tested (20/21) had highly IgA-coated *Rt* in fecal samples. The PBMC from four of these 20 patients (20%), who also had responses to the *Am* peptides, reacted with the nearly identical HLA-presented *Rt*-HSP70 peptide (R1 peptide, **Figure 5A**, right) and often with the two additional predicted *Rt*-HSP70 peptides (R2–R3 peptides) and corresponding human mimic peptides (H6, H8, and H9 peptides; **Figure 5B**, right). The PBMC from the one RA patient who lacked fecal *Rt* IgA coating, but who had highly IgA-coated *Am* in stool, had a positive T cell response to one *Rt*-derived peptide but not to a corresponding human mimic peptide. No reactivity to *Rt* peptides was detected in PBMC from HC subjects (data not shown).

Thus, four of 18 RA patients (22%) with highly IgA-coated *Am* in stool samples had consistently positive T cell responses with *Am*-SCS- and *Am*-cDnaK-derived peptides, often with *Rt*-HSP70 peptides and corresponding human mimic peptides, a result suggestive of T cell epitope mimicry and epitope spreading. In contrast, the PBMC from the three RA patients who had negative IgA-coating of fecal *Am* as well as HC subjects showed no reactivity with the *Am* peptides.

### IgA and IgG antibody responses to citrullinated *Am, Rt,* and human mimic proteins

We next determined serum IgG and IgA antibody responses to native and citrullinated recombinant *Am*-cDnaK (cit-*Am*-cDnaK), *Rt*-cDnaK (cit-*Rt*-cDnaK), and human HSPA5 (cit-HuHSPA5) proteins in RA patients compared with that in patients with osteoarthritis (OA) or spondyloarthropathy (SpA) and in healthy control (HC) subjects. In RA patients, a single sample taken close to the date of the T cell sample was tested first. Recombinant preparations of the *Am*- and *Rt*-cDnaK proteins were generated without difficulty, but the membrane spanning domains of the *Am*-SCS protein prevented the generation of a suitable recombinant protein for testing. A positive response was defined as ≥2 SD above the mean value in healthy control subjects.

Of the 21 RA patients, four patients (19%) had positive IgG antibody responses to cit-*Am*-cDnaK, three of whom had higher responses to the citrullinated protein than to the native uncitrullinated protein, a result suggestive of reactivity with citrullinated B cell epitopes (**Figure 6A**, left). The RA patient with the highest IgG response also had IgA reactivity with cit-*Am*-cDnaK (**Figure 6A**, right). One patient with OA, two patients with SpA, and two HC subjects also had IgG or IgA responses to native *Am*-cDnaK, but their reactivity was less to the citrullinated protein, suggesting that reactivity was only to the uncitrullinated protein (**Figure 6A**). Additionally, IgG or IgA antibody responses to citrullinated *Rt*-cDnaK or Hu-HSPAS were detected in one other RA patient and two HC subjects, but the responses were comparable to or lower than the responses to the equivalent native protein (**Figures 6B, C**). Thus, clear examples of responses to citrullinated proteins were found only to cit-*Am*-cDnaK in four RA patients and not in the comparison groups.

**Figure 6:**
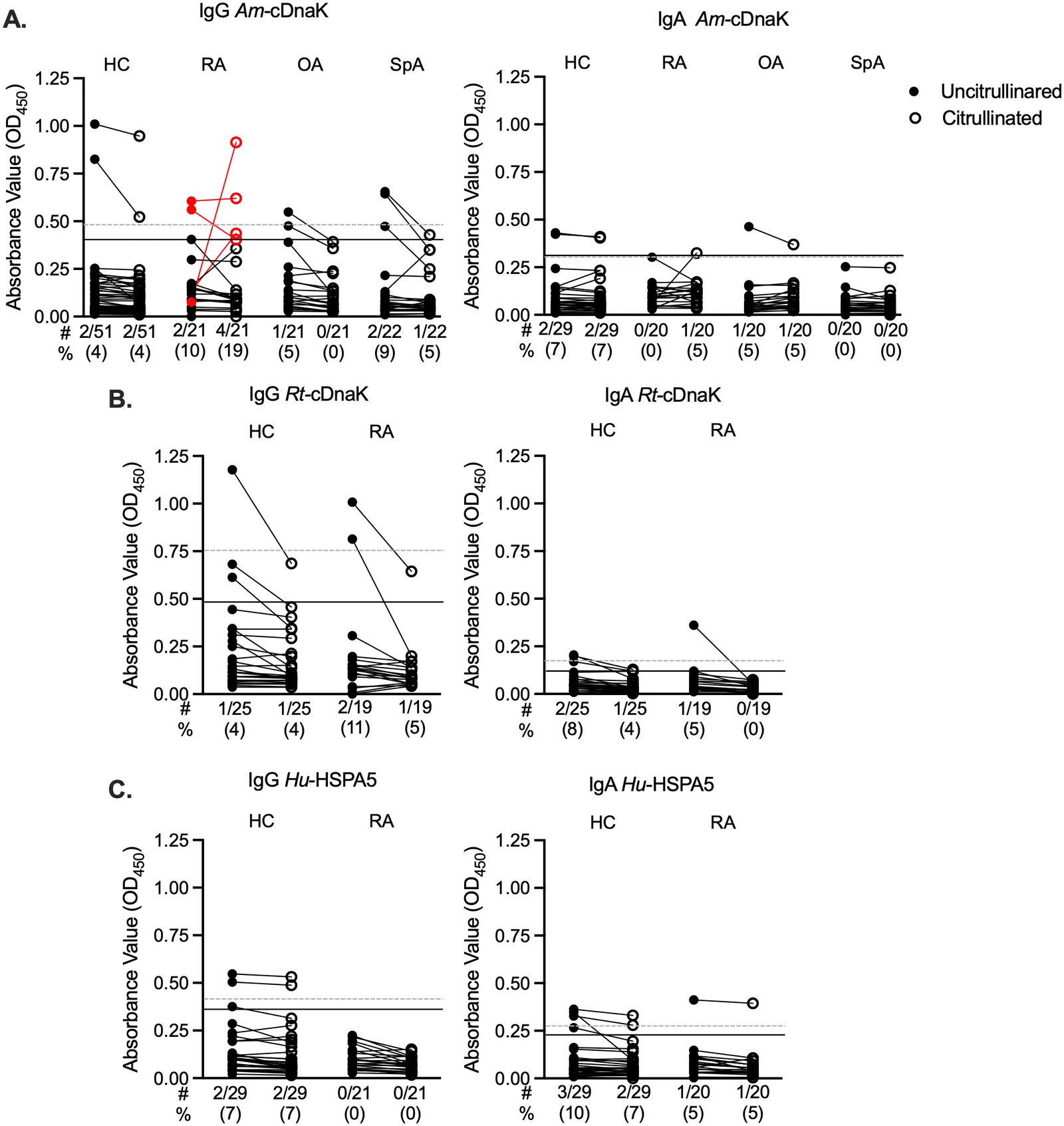
IgG and IgA antibody responses to uncitrullinated and citrullinated microbial and human mimic proteins. Serum IgG and IgA antibody responses to uncitrullinated (closed circles) and citrullinated (open circles) *Am*-cDnaK protein were determined by ELISA in 21 patients with RA, 21 with osteoarthritis (OA), 22 with spondyloarthropathy, and 51 healthy control subjects (HC) **(A)**. In RA patients and HC with remaining sera, IgG and IgA antibody responses were also determined for uncitrullinated and citrullinated *Rt*-cDnaK **(B)** and Hu-HSPA5 **(C)**. The dashed gray line represents 2 SD above the mean value of HC subjects for the uncitrullinated protein, and the solid black line represents 2 SD above the mean value of the HC subjects for the citrullinated protein. The red dots identify antibody responses in the four RA patients who had IgG reactivity to citrullinated *Am*-cDnaK that was greater than or equal to 2 SD above the mean value of the HC cohort.

In the 21 patients, serial serum samples were available in one with pre-RA, five with early RA, and five with chronic RA, which were then tested for reactivity with the microbial and human proteins. Compared with the initial testing of one sample per patient, a greater number of patients had positive responses with testing of serial samples. Five of the 11 patients had IgG antibody responses to cit-*Am*-cDnaK in at least one of the dates tested, which were close to or higher than 2 SD above the mean value in healthy control subjects (**Figure 7A**). In four of the five patients, these responses occurred during pre-RA or soon after transition to clinical RA, and in one patient they occurred years after disease onset during two flares of arthritis. Two of the five patients who had responses to cit*-Am-*cDnaK also had low-level reactivity with its human mimic protein, cit-HuHSPA5 (**Figure 5B**). Finally, two additional patients had weak reactivity with cit-HuHSPA5 but lacked responses to cit*-Am-*cDnaK.

**Figure 7:**
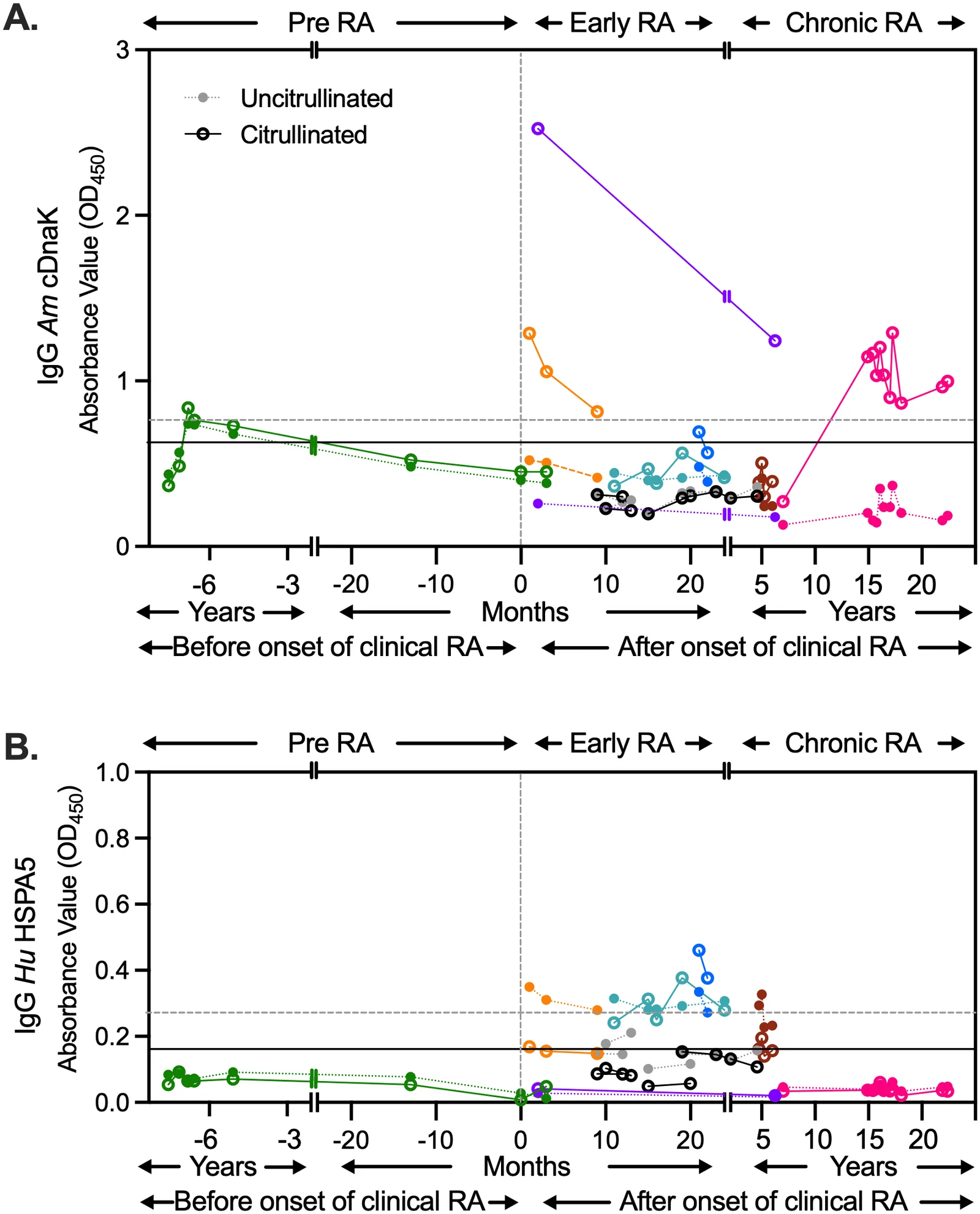
Longitudinal IgG antibody responses to uncitrullinated and citrullinated *Am*-cDnaK and Hu-HSPA5 proteins in RA patients. IgG antibodies against uncitrullinated (closed circles with dashed lines) and citrullinated *Am*-cDnaK (open circles with solid lines) **(A)** or to uncitrullinated (closed circles with dashed lines) and citrullinated Hu-HSPA5 (open circles with solid lines) **(B)** were measured in 11 patients from whom serial serum samples were available. The HLA-DR alleles of five patients with positive IgG responses to citrullinated *Am-*cDnaK, two of whom had positive responses to Hu-HSPA5, were as follows: green—DRB1*1104/1301, orange—*0101/0101, purple—*0301/1302, blue—*0401/0404, and pink—*0101/0401. The dashed gray line represents 2 SD above the mean value for healthy control subjects for the uncitrullinated protein, and the solid black line represents 2 SD above the mean value for 24 healthy control subjects for the citrullinated protein.

### Summary of T and B cell data

Of 21 total patients, two had T cell responses to *Am*-SCS, *Am-*cDnaK, and HuHSPA5/HuHSPA9 mimic peptides in addition to IgG antibody responses to cit*-Am-*cDnaK and, in one, to cit-HuHSPA5. Two additional patients had T cell reactivity to *Am*-SCS and *Am-*cDnaK and responses to either HuHSPA5 or HuHSPA9 peptides but lacked antibody responses to these proteins. Four additional patients had only IgG antibody responses to cit*-Am-*cDnaK, and two also had weak antibody reactivity to cit-HuHSPA5. Altogether, eight of 21 patients (38%) had T cell responses to the *Am*-cDnaK peptide and a human mimic peptide (Hu-HSPA5) and/or IgG antibodies to citrullinated forms of these proteins.

### Comparison of clinical findings according to systemic immune responses to *Am-*cDnaK

The clinical findings in the eight RA patients who had *Am*-cDnaK responses were compared with those in the 13 patients who lacked such responses (**Table 2**). Typical of RA cohorts (20), both groups had similar median ages, sex ratios favoring women, and frequencies of SE alleles. Moreover, in our patient cohort, both groups had close to the same frequencies of patients with highly IgA-coated *Am* in stool samples. Despite the small numbers, the group with systemic *Am* immune responses trended toward higher percentages of patients with new-onset RA (62.5% vs. 38.5%, *P* = 0.38), ACPA and RF (50% vs. 15.4%, *P* = 0.15), and higher CRP levels (median value, 12.2 vs. 3.4, *P* = 0.2). Finally, the Disease Activity Score (DAS) was calculated based on the examination of 28 joints, which were ranked as normal, tender, or swollen, and a CRP or ESR value (26). Perhaps because most patients were already taking DMARDs, which would dampen inflammatory responses; the median was only slightly higher in the group with systemic *Am* immune responses.

**Table 2:** Demographics and clinical findings in RA patients according to T cell reactivity to *Am* epitopes and/or B cell responses to citrullinated *Am*-cDnaK.

Characteristics	T and/or citrullinated B cell reactivity to *Am*^a^	No T and/or citrullinated B cell reactivity to *Am*	
	Group 1 (*N* = 8)	Group 2 (*N* = 13)	*P*-value^b^
Demographics			
Age, median (range)	53.5 (24–66)	51 (18–74)	0.71
Sex, female/male	6/2	10/3	1.00
RA stage			
Pre-RA, no. of patients (%)	1 (12.5)	0 (0.0)	0.38
New onset, no. patients (%)	5 (62.5)	5 (38.5)	0.38
Chronic, no. patients (%)	2 (25.0)	8 (61.5)	0.18
HLA-DRB1 alleles			
Shared epitopes^c^, no. of patients (%)			
1	3 (37.5)	6 (46.2)	1.00
2	3 (37.5)	2 (15.4)	0.33
None	2 (25.0)	5 (38.5)	0.66
Fecal *Am* immunity (ICI > 0.7) (%)	7 (87.5)	11 (84.6)	1.00
RA autoantibodies			
ACPA (>15.6), no. positive (%)	6 (75.0)	6 (46.2)	0.37
RF (>30), no. positive (%)	5 (62.5)	3 (23.1)	0.16
ACPA and RF (%)	4 (50.0)	2 (15.4)	0.15
Inflammatory markers			
CRP, mg/L, median [95% CI]	12.2 [0.7, 60.6]	3.4 [1.4, 7.0]	0.20
ESR, mm/h, median [95% CI]	17.5 [5.0, 36.0]	10.0 [7.0, 17.0]	0.42
Disease Activity Score			
DAS CRP, median [95% CI]	2.5 [1.4, 3.9]	1.9 [1.7, 2.9]	0.51
DAS ESR, median [95% CI]	2.7 [1.4, 4.1]	2.4 [1.5, 3.0]	0.46

RA, rheumatoid arthritis; ACPA, anti-citrullinated protein antibodies; RF, rheumatoid factor; CRP, C-reactive protein; ESR, erythrocyte sedimentation rate; DAS, Disease Activity Score.

^a^Two patients had T cell responses to *Am*-cDnaK epitopes and B cell reactivity with citrullinated *Am*-cDnaK protein, two had T cell responses to *Am*-cDnaK epitopes alone, and four had reactivity with citrullinated *Am*-cDnaK protein alone.

^b^None of the comparisons were statistically significant.

^c^Alleles 0101, 0102, 0401, 0404, 0405, 0408, and 1001.

### Sensitivity analysis

A preliminary sensitivity analysis showed that one patient with pre-RA, five of 10 patients with early RA, and only two of 10 patients with chronic RA had T cell responses to the *Am*-cDnaK peptide and a human mimic peptide (Hu-HSPA5) and/or IgG antibodies to citrullinated forms of these proteins. Thus, there was a suggestion (not statistically significant) that systemic immune responses to *Am*-cDnak may occur around the time of transition from pre-RA to clinical RA. However, the number of patients who had reactivity with the human mimic HSPA5 protein was not large enough to determine when molecular mimicry may have first developed to the human mimic protein.

## Discussion

In the current study, most RA patients had many highly IgA-coated bacterial taxa in stool samples. Because the patients’ PBMC and serum samples were limited, we selected two of the most common highly IgA-coated taxa for initial study. In our RA cohort, 88% had highly IgA-coated *Am* and 94% had highly IgA-coated *Rt*. Although tested by different methods at an earlier time, the percentage of highly IgA-coated *Am* appears to be less in healthy individuals (~55%) than in RA patients (23). These percentages may be influenced by age, sex, diet, and geographic location, which were not matched in our case and control study cohort. However, the purpose of identifying highly IgA-coated commensals in our study was not to compare groups but to identify candidate highly IgA-coated bacterial taxa that may trigger pathogenic immune responses in RA patients.

IgA, which is the predominant antibody isotype produced at mucosal surfaces, is a critical mediator of intestinal immunity (27). High IgA-binding marks the likely presence of potential disease-causing bacteria in the mucosal layer of the bowel (23). Both *Am* and *Rt* colonize the mucosal barrier and degrade intestinal mucin. However, *Am* is unusual in that it both degrades and produces mucin, which may be beneficial or detrimental depending on the circumstances (28, 29). Mucin degradation disrupts the integrity of the intestinal mucosal barrier (30–33), which allows binding of the organisms to the intestinal mucosa, leading to mucosal inflammation and potentially to the hematogenous spread of organisms to other sites. This sequence of events has been documented for an adherent strain of *E. coli* in patients with arthritis-associated Crohn’s disease (34). In addition, increased intestinal permeability allowing the passage of antigens from the intestinal flora has been noted in several autoimmune diseases, including type 1 diabetes, multiple sclerosis, and rheumatoid arthritis (35, 36).

*Am* and *Rt* have been implicated in several diseases (32, 37). Multiple studies have linked an increased burden of *Rt* with inflammatory bowel disease (IBD) (33, 38, 39) or with colorectal cancer (40). In addition, *Am* has increased abundance in the gut microbiota of patients with multiple sclerosis (MS) (29, 37, 41). In MS patients, autoreactive CD4+ T cell clones cross-reacted with HLA-DR-presented *Am* peptides and human mimic peptides derived from myelin (42), and elevated IgG antibodies to *Am* lysates were detected in cerebrospinal fluid (43, 44). Conversely, reduced *Am* abundance has been linked to several metabolic disorders, including obesity, type 2 diabetes, and hypertension (28, 45, 46), leading to the development of *A. muciniphila* supplements as a possible first-generation probiotic for the treatment of these disorders (47).

The systemic spread of commensal microbes or their remnants to joints is not a requisite part of the “mucosal origins hypothesis”. However, microbes in the mucosal layer of the gut commonly spread systemically (36) but cause disease only in individuals with certain genetic or environmental risk factors (23). In RA patients, staining of bacterial peptidoglycan has been detected in the synovial tissue (48), and within synovia, some antigen-presenting cells, primarily macrophages and dendritic cells, contained bacterial peptidoglycan and expressed proinflammatory cytokines (49). In our previous studies, an HLA-DR-presented peptide of *P. copri* (Pc-p27) was identified from the PBMC of one RA patient using nano-LC-MS/MS (11), and in three of 18 patients (17%) *Prevotella* DNA was detected in synovial fluid by PCR. Taken together, these findings are consistent with the hematogenous spread of certain commensal microbes or their remnants from the bowel to the joints (16), which may facilitate RA development. Unfortunately, synovial fluid samples were not available for PCR studies in the current patients.

We adopted the same methodology for the current study. Using nano-LC–MS/MS, two HLA-DR-presented *Am* peptides (*Am*-SCS and *Am*-cDnaK) were identified directly from synovial tissue or SFMC. Although the synovial-identified *Am*-cDnaK epitope has close sequence homology with an epitope of a human mimic heat shock protein, we think that these were *Am*-derived epitopes. First, both the *Am*-SCS and *Am*-cDnaK HLA-DR-presented peptides had 100% homology with the two synovial-identified *Am* peptides. Second, patients who had reactivity with the synovial HLA-DR-presented peptides often had responses to other predicted *Am*-SCS and *Am*-cDnaK T cell epitopes. Third, in a subset of our RA patients, these *Am* peptides stimulated T cell responses, as assessed in IFNγ ELISpot assays, and antibody responses were found to the citrullinated *Am* proteins from which these peptides were derived. We would not expect such results if human HLA-DR-presented peptides in synovium had been erroneously assigned as microbial-derived peptides.

Focusing on the evaluation of T and B cell responses to one *Am* protein, eight of 21 patients (38%) had T cell responses to the *Am*-cDnaK peptide and a human heat shock protein-mimic peptide (Hu-HSPA5) and/or IgG antibodies to citrullinated forms of these proteins. This percentage is consistent with our previous finding of 41 of 127 RA patients (32%) who had IgG or IgA antibody responses to a single *P. copri* protein (*Pc*-p27) identified by nano-LC–MS/MS (11). The percentage of RA patients who have reactivity with *S. didolesgii*, the other bowel commensal currently implicated in RA pathogenesis, is not yet known (12).

T cell epitope mimicry between bacterial and human heat shock proteins (HSP), which are highly conserved from bacteria to mammals, has been implicated previously in RA (50–52). Using a murine model, Van Herwijnen et al. identified a disease-relevant epitope of a *M. tuberculosis*-derived DnaK protein and a human mimic epitope, mB29a, the latter of which stimulated T cell proliferation and pro-inflammatory cytokine production from the PBMC of patients with RA or psoriatic arthritis or from HC subjects (53, 54). Conversely, tolerogenic dendritic cells (DCs) from RA patients pulsed with human mB29a promoted regulatory T cell responses (54), and therefore tolerogenic DCs are now being tested in phase 1 and 2 trials as a potential treatment for RA (51). Molecular mimicry between other microbial and human proteins has also been shown to have a pathogenic role in several other rheumatic diseases, including antiphospholipid syndrome (55) and systemic lupus erythematosus (56–58).

ACPA are found in approximately 60%–80% of RA patients and rarely in patients with other diseases (59). Additionally, peripheral blood plasmablasts, some of which reacted with the periodontal pathogens *Porphyromonas gingivalis* or *Aggregatibacter actinomycetemcomitans* or with Epstein–Barr virus (EBV), have been reported to promote ACPA production (9, 60, 61). Furthermore, *A. actinomycetemcomitans* was demonstrated to induce dysregulated neutrophil hypercitrullination of proteins targeted by ACPA via the production of a bacterial pore-forming toxin, leukotoxin A (9). Conversely, *P*. *gingivalis* was shown to express a unique PAD enzyme capable of citrullinating both bacterial and host RA candidate autoantigens, including fibrinogen, alpha-enolase, vimentin, and histones (62). Moreover, ACPA against human HSP have been noted in RA patients, but they are not as frequent or prominent as ACPA to fibrinogen, α-enolase, or vimentin (50, 63–65). Consistent with previous reports (52, 66–69), a few patients in our study with OA or SpA as well as HC subjects had antibody responses to native, uncitrullinated *Am*-cDnaK and/or Hu-HSPA5, but only RA patients had responses to the citrullinated forms of these proteins. We did not find unequivocal antibody responses to cit-*Rt*-DnaK in RA patients. Although the T cell epitopes of *Am*-cDnaK and *Rt*-cDnaK are nearly identical, the two proteins have only 59% sequence homology (Uniprot database), which may explain differences in antibody cross-reactivity to cit-*Rt*-cDnaK. This example shows that T cell epitope mimicry between a microbial and human protein may not necessarily lead to pathogenic ACPA.

We present here hypotheses and future directions that may help explain the mechanisms by which *Am* may be involved in RA pathogenesis. First, *Am*, which is highly IgA-coated in most RA patients, may bind regularly to gut mucosa, spread hematogenously, and undergo phagocytosis by circulating monocytes and macrophages. This would not usually cause disease, but in genetically susceptible individuals, immune responses to *Am* in the mucosa or systemically may induce the development of ACPA and RF, which may increase over time. Occasionally, however, *Am* or its components, perhaps within cells, may spread to the joints. Although this step may not be necessary for joint inflammation in all patients, individuals at risk for RA exhibit dysregulated proinflammatory immune responses prior to and during transition to active disease (14). This appears to be the time when one finds the highest levels of *Am* immune responses, which may enhance or ignite joint inflammation. Thus, an immune response to the microbial components in the joints might explain why they have an intense inflammatory response there, but apparently not in the intestinal mucosa.

Furthermore, it will be important to determine the balance between proinflammatory and anti-inflammatory features of immune responses to the implicated microbes, as shown by cytokine profiles, T effector and Treg cell ratios, and glycosylation profiles of citrullinated antibodies. Within this proinflammatory milieu, epitope mimicry and epitope spreading in genetically susceptible individuals may allow the further development of autoimmunity to a range of citrullinated human proteins, including fibrinogen, alpha-enolase, vimentin, and histones (12). In addition to peptide binding to SE alleles, interactions between these alleles and specific nucleotide polymorphisms in genes such as *PTPN22*, *HTR2A*, and *MAP2K4* have also been reported to influence the overall risk of RA (70–72). With DMARD therapy, inflammation lessens, and partial or complete tolerance to *Am* may be restored. However, this entire scenario may recur in patients with established RA, resulting in flares of arthritis.

Our study had several limitations. First, the sample size was small; only two highly IgA-coated commensal flora, *Am* and *Rt*, and only two *Am* proteins, a transporter and heat shock protein, were evaluated. Additional *Am* proteins, particularly certain outer-surface proteins, may be considerably more antigenic than the transporter and chaperone proteins studied here. Second, RA patients have many other highly IgA-coated gut commensal flora; it will be important to explore immune responses to additional flora for a possible role in RA pathogenesis. Third, linking immunity to *Am* proteins in the joints was based on the identification of HLA-DR presentation of *Am* peptides in the joints; joint fluid samples were not available to search for bacterial peptidoglycan or *Am* DNA by PCR. Fourth, we identified only HLA-DRB1 alleles, but certain DQ alleles are also strongly associated with RA (73). Fifth, a normal control population was not developed for comparisons with the commensal flora of RA patients (**Figure 2**), and the healthy control populations used for T and B cell studies were not matched for age, sex, or HLA-DR types (**Table 2**). Sixth, because of the small sample size, comparisons of patient subgroups often showed only trends rather than significant differences. Therefore, the associations found here may be altered with the testing of larger numbers of patients and matched control populations. Seventh, there was a trend toward the development of cit-*Am*-cDnaK immune responses primarily during pre-RA and early RA, but the number of patients who had reactivity with the human mimic HSPA5 protein was not large enough to determine when molecular mimicry first developed to the human mimic protein.

Notwithstanding these limitations, our study was meant to be a hypothesis-generating evaluation of a relatively small number of patients. We found that most RA patients had many highly IgA-coated bacterial taxa in stool samples, commonly including *Am* and *Rt* (primary outcome). The HLA-DR-presentation of *Am* and *Rt* peptides derived from a *Am*-cDnaK chaperone protein could be identified in several RA patients’ synovial tissue, and T and B cell responses to these microbial and human mimic proteins were found in a minority of the study patients (secondary supporting outcomes). Thus, our preliminary study implicates *Am* as a candidate gut commensal microbe having a possible role in RA pathogenesis and provides an additional hypothesis for exploration in how commensal organisms may initiate or enhance autoimmune joint inflammation.

## Data Availability

Raw sequences for the data presented in **figure 2** are available at the National Center for Biotechnology Information Sequence Read Archive under the BioProject accession number PRJNA1531587. The mass spectrometry proteomics data presented in **figure 3** have been deposited to the ProteomeXchange Consortium via the PRIDE partner repository with the dataset identifier PXD084172. All other data supporting the conclusions of this article will be made available by the authors, without undue reservation.
